# Interplay Between Oxidative Stress and Inflammation in Aquatic Animals: Mechanisms, Consequences, and Implications for Aquaculture Health

**DOI:** 10.3390/antiox15020208

**Published:** 2026-02-04

**Authors:** Zi-Yan Liu, Yang Yu, Xiao-Zheng Yu

**Affiliations:** 1 Key Laboratory of Pollution Exposure and Health Intervention of Zhejiang Province, College of Biological and Environmental Engineering, Zhejiang Shuren University, Hangzhou 310015, China; 2 State Key Laboratory of Biocontrol, Guangdong Province Key Laboratory for Aquatic Economic Animals, Guangdong Provincial Engineering Technology Research Center for Healthy Breeding of Important Economic Fish, School of Life Sciences, Sun Yat-Sen University, Guangzhou 510275, China

**Keywords:** aquaculture, pathogens, reactive oxygen species, immune regulation, sustainable aquaculture

## Abstract

Oxidative stress and inflammation are two tightly interconnected processes that shape the physiological and pathological responses of aquatic animals to environmental and pathogenic challenges. Reactive oxygen species (ROS) act as key molecular mediators linking oxidative damage with immune activation, forming a bidirectional amplification loop in which oxidative stress triggers inflammation, and inflammation further enhances ROS production. This vicious cycle disrupts immune homeostasis, damages vital organs such as the liver, intestine, and gills, and ultimately increases disease susceptibility in aquaculture species. Recent studies have revealed that breaking this ROS–inflammation loop through integrated strategies, combining antioxidant enhancement, inflammation modulation, and immune regulation, can significantly improve stress tolerance and survival. Particularly in viral diseases, targeting the ROS–inflammation–virus replication axis has emerged as a promising approach for effective control. This review systematically summarizes the mechanistic crosstalk between oxidative stress and inflammation, highlights their biological consequences, and proposes mechanism-based prevention strategies for sustainable aquaculture health management.

## 1. Introduction

Oxidative stress and inflammatory responses represent two tightly interconnected biological processes that exert profound influence on the physiological status of living organisms. These processes do not occur independently but are coordinated through complex networks of signaling molecules and regulatory pathways. Oxidative stress arises when the balance between the generation of reactive oxygen species and the antioxidant defense system is disrupted, leading to oxidative damage and altered cellular signaling. Inflammation, on the other hand, is a fundamental immune reaction that protects organisms against pathogens and injury but can become detrimental when excessively activated or chronically sustained. Increasing evidence suggests that oxidative stress and inflammation are reciprocally regulated and often amplify each other, forming a self-perpetuating loop that underlies a wide range of pathological and adaptive processes [1,2,3,4].

In aquatic animals, the interaction between oxidative stress and inflammatory responses plays an essential role in stress adaptation, maintenance of immune homeostasis, and modulation of disease susceptibility. Environmental fluctuations, aquaculture density, nutritional imbalance, and pathogen exposure can all trigger redox imbalance and immune activation [5,6,7,8,9]. The intricate crosstalk between redox signaling and inflammatory pathways determines how fish, crustaceans, and mollusks respond to stressors and maintain physiological stability. Understanding this interrelationship is therefore critical for improving animal health, enhancing resilience to environmental stress, and developing targeted interventions in aquaculture systems.

The present review aims to provide a comprehensive overview of the bidirectional relationship between oxidative stress and inflammation in aquatic organisms. It first clarifies their conceptual foundations and explores how molecular interactions between redox mediators and inflammatory regulators form dynamic feedback loops. The review further discusses the biological consequences of this interplay at cellular and systemic levels and evaluates its implications for aquaculture health management and disease prevention. Unlike previous studies that have treated oxidative stress or inflammation as separate entities, this article integrates both processes within a unified mechanistic framework. It emphasizes how their convergence shapes immune function, stress tolerance, and growth performance in cultured species. By combining recent advances in redox biology, immuno-physiology, and aquaculture science, this work provides new insights into the mutual regulation of oxidative and inflammatory responses. Moreover, it highlights practical strategies that exploit this relationship to enhance disease resistance and promote sustainable aquaculture. Through this integrated perspective, the review offers not only an updated synthesis of current knowledge but also a conceptual foundation for future studies aiming to strengthen the health and welfare of aquatic animals.

## 2. Core Concepts: Foundational Mechanistic Framework

### 2.1. Oxidative Stress

Oxidative stress represents a fundamental disturbance in the redox equilibrium within biological systems. It occurs when the production rate of reactive oxygen species (ROS) such as superoxide anion (O_2_^−^), hydrogen peroxide (H_2_O_2_), and hydroxyl radicals (•OH), together with reactive nitrogen species (RNS), exceeds the neutralizing capacity of the antioxidant defense network. This antioxidant system is composed of both enzymatic components, including superoxide dismutase (SOD), catalase (CAT), and glutathione peroxidase (GPx), and non-enzymatic antioxidants such as glutathione (GSH), vitamins C and E, and carotenoids. When the delicate balance between oxidation and reduction processes is disrupted, oxidative stress emerges as a biochemical state characterized by excessive free radical accumulation, oxidative damage to cellular macromolecules, including DNA, proteins, and lipids, and the initiation of redox-sensitive signaling cascades that may alter cellular metabolism and viability [10,11].

In aquatic animals, oxidative stress is a common physiological challenge due to the highly variable and often harsh conditions encountered in aquaculture environments [12,13]. Multiple external and internal stressors can trigger ROS overproduction and compromise antioxidant defenses. Environmental stressors such as elevated ammonia and nitrite concentrations, heavy metal exposure, salinity and temperature fluctuations, and hypoxia can induce oxidative burden by disrupting mitochondrial function and promoting electron leakage [12,14,15,16]. High-density culture conditions exacerbate this effect by reducing dissolved oxygen levels and accumulating metabolic wastes, thereby intensifying oxidative challenges [17]. In zebrafish, exposure to cyanobacterial aphantoxins markedly increased ROS and MDA levels while depleting GSH in the brain, indicating enhanced lipid peroxidation and redox imbalance, accompanied by compensatory activation of antioxidant enzymes such as SOD, CAT, and GPx [18]. Similarly, in Pacific white shrimp (*L. vannamei*), cold shock and air exposure during waterless transportation induced transient ROS accumulation and elevated MDA levels, triggering tissue-specific antioxidant responses in the hepatopancreas, highlighting lipid peroxidation as a key indicator linking oxidative stress to cellular stress responses [19]. Moreover, pathogen invasion, including bacterial, viral, and parasitic infections, can stimulate a respiratory burst in phagocytic cells, resulting in elevated ROS production as part of the innate immune response in fish [20]. However, when this oxidative response is insufficiently counterbalanced by antioxidant defenses, excessive ROS may exacerbate oxidative damage and inflammatory pathology, as further discussed in Section 3.2. Nutritional imbalance, particularly deficiencies in antioxidant nutrients such as selenium, vitamin E, or carotenoids, further weakens the redox buffering system and makes cultured species more vulnerable to oxidative injury [21,22,23]. Collectively, these factors contribute to a persistent state of oxidative challenge that can compromise immune competence, growth performance, and overall health in aquaculture species.

### 2.2. Inflammation

Inflammation is a highly conserved defense mechanism that enables organisms to recognize and respond to harmful stimuli. At its core, inflammation is triggered by the detection of danger signals, including pathogen-associated molecular patterns (PAMPs) derived from invading microorganisms and damage-associated molecular patterns (DAMPs) released from injured host cells [24]. These molecular cues are recognized by pattern recognition receptors (PRRs) such as Toll-like receptors and NOD-like receptors, which subsequently activate innate and adaptive immune responses [25,26]. The inflammatory cascade involves the recruitment and activation of immune effector cells, including macrophages, neutrophils, and lymphocytes, that release an array of signaling molecules. These include pro-inflammatory cytokines such as tumor necrosis factor-alpha (TNF-α), interleukin-1 beta (IL-1β), and IL-6, as well as anti-inflammatory mediators like IL-10 and transforming growth factor-beta (TGF-β). Additional inflammatory mediators, including prostaglandins, leukotrienes, and histamine, modulate vascular permeability, leukocyte migration, and tissue repair, thereby orchestrating a coordinated defense response.

However, inflammation exhibits a dualistic nature. When appropriately regulated, it serves as a vital protective mechanism that facilitates the clearance of pathogens, removal of damaged cells, and restoration of tissue homeostasis. Conversely, excessive or chronic inflammatory activation can become detrimental. In aquatic animals, uncontrolled inflammation has been associated with tissue necrosis, disruption of epithelial barriers such as the intestinal mucosa, hepatocellular injury, and systemic immune suppression [27,28,29]. Understanding this delicate balance between protective and pathological inflammation is therefore critical for elucidating how immune homeostasis is maintained or disrupted under aquaculture conditions.

Together, oxidative stress and inflammation form the mechanistic foundation for many stress-related disorders and disease processes in aquatic animals. Their close interdependence, mediated through shared signaling pathways and redox-sensitive transcription factors, represents a pivotal axis of physiological regulation. Deciphering the molecular principles underlying this oxidative–inflammatory interface not only advances our understanding of aquatic immuno-physiology but also provides theoretical guidance for developing nutritional, environmental, and genetic strategies to enhance resilience and health in cultured species.

## 3. Bidirectional Regulation Mechanisms: The Mutual Activation Loop Between Oxidative Stress and Inflammation

Oxidative stress and inflammation are not independent biological events but are intricately linked through a web of molecular interactions and feedback amplification loops. They share common signaling molecules and regulatory pathways that mutually activate and reinforce each other, leading to a self-perpetuating cycle of cellular damage and immune dysregulation. The interplay between these processes forms a mechanistic mutual activation loop, which can be viewed as both a physiological defense strategy and a pathological amplifier when uncontrolled.

### 3.1. Oxidative Stress as an Upstream Driver of Inflammation

Reactive oxygen species (ROS) act as crucial oxidative signaling molecules that initiate and intensify inflammatory responses through several key molecular mechanisms. One major pathway involves the activation of the transcription factor nuclear factor kappa B (NF-κB) [30,31,32]. Under basal conditions, NF-κB remains inactive in the cytoplasm by forming a complex with its inhibitory protein IκB. Excessive ROS oxidize critical cysteine residues on IκB, marking it for ubiquitin-dependent degradation. The released NF-κB then translocates into the nucleus, where it binds to specific promoter regions to induce the transcription of proinflammatory genes such as tumor necrosis factor-alpha (TNF-α), IL-1β, IL-6, cyclooxygenase-2 (COX-2), and inducible nitric oxide synthase (iNOS). These mediators further amplify the inflammatory response. Empirical evidence supports this mechanism in aquatic species. For example, viral infection has been shown to induce ROS accumulation and alter intracellular redox homeostasis, thereby regulating viral replication through NF-κB signaling [33]. In fish EPC cells infected with spring viraemia of carp virus (SVCV), ROS levels increased in a time-dependent manner, and modulation of redox status directly affected viral replication; enhanced ROS production activated NF-κB and suppressed SVCV replication, whereas antioxidant treatment reduced ROS and promoted viral proliferation, highlighting a ROS–NF-κB-dependent anti-viral and anti-inflammation mechanism. Similarly, nutritional stress-induced oxidative imbalance has been linked to inflammatory activation via NF-κB. Phenylalanine deficiency in grass carp led to elevated ROS levels, impaired antioxidant capacity, and activation of NF-κB–associated pro-inflammatory cytokines (including IL-1β, IL-8, and TNF-α), concomitant with downregulation of Nrf2 signaling and disruption of epithelial barrier integrity [34]. In immune regulation, prolactin stimulation in gilthead seabream leukocytes induced ROS production and pro-inflammatory cytokine expression, effects that were abolished by inhibitors of Jak/Stat and NF-κB pathways, indicating a coordinated regulation of ROS generation and NF-κB-mediated immune activation [35]. Moreover, pharmacological inhibition studies in LPS-stimulated zebrafish embryos demonstrated that suppression of either ROS or NF-κB markedly attenuated inflammatory mediator production, confirming that ROS act upstream of NF-κB in inflammation induction [31]. Interestingly, oxidative stress-induced NF-κB activation can be effectively alleviated by antioxidant or functional compounds in fish. Resveratrol supplementation markedly attenuated LPS-induced hepatic inflammation and oxidative stress in gibel carp (*Carassius gibelio*) by inhibiting NF-κB signaling, thereby reducing ROS accumulation and inflammation [36]. Similarly, ferulic acid supplementation significantly alleviated gill inflammation and oxidative damage caused by chronic difenoconazole exposure in freshwater carp by suppressing ROS accumulation, inhibiting NF-κB activation, and restoring redox homeostasis [37]. Collectively, these studies underscore that ROS serve as pivotal signaling molecules driving inflammatory responses in aquatic organisms predominantly via NF-κB activation, while modulation of redox balance represents an effective strategy to mitigate inflammation and tissue injury under pathogenic, nutritional, or environmental stress conditions.

A second major mechanism involves the activation of the NLRP3 inflammasome, a cytosolic multiprotein complex that mediates the maturation of inflammatory cytokines. ROS can directly damage mitochondria, leading to the release of mitochondrial DNA (mtDNA) into the cytoplasm. The presence of mtDNA and oxidized mitochondrial components acts as damage-associated molecular patterns (DAMPs) that activate the NLRP3 inflammasome. Activated NLRP3 recruits and activates caspase-1, which processes pro–IL-1β and pro–IL-18 into their mature forms. These cytokines are subsequently released during pyroptosis, a form of inflammatory programmed cell death, causing strong local inflammation and systemic immune activation [37]. Accumulating evidence indicates that, in aquatic animals, ROS serve as critical upstream signals that drive inflammatory responses, predominantly through activation of the NLRP3 inflammasome. In common carp (*Cyprinus carpio* L.), NLRP3 is evolutionarily conserved and widely expressed in immune-related tissues, where it can assemble into functional inflammasome complexes in response to bacterial and viral infections, thereby inducing caspase activation, pyroptosis, and pro-inflammatory cytokine release [38]. Multiple studies have demonstrated that excessive ROS production is a key trigger for NLRP3 inflammasome activation. For instance, oxidative stress induced by environmental pollutants such as perfluorooctane sulfonate (PFOS) activates the ROS–NLRP3 axis, leading to enhanced IL-1β production, pyroptosis, and metabolic disorders in fish hepatocytes, effects that can be effectively alleviated by ROS scavengers or pharmacological inhibition of NLRP3 in grass carp hepatocytes [39]. Similarly, chronic difenoconazole exposure induces ROS accumulation and activates NF-κB-dependent NLRP3 inflammasome signaling in carp gills, resulting in inflammatory injury, whereas antioxidant intervention effectively suppresses this pathway [37]. In addition to chemical stressors, nutritional and metabolic stress, such as excessive palmitic acid exposure, promotes mitochondrial dysfunction and ROS overproduction, which subsequently activates the NLRP3 inflammasome via NF-κB priming and impaired AMPK-mediated mitophagy, ultimately driving IL-1β-mediated inflammation in large yellow croaker (*Larimichthys crocea*) [40]. Conversely, immunomodulatory and antioxidant factors, including IL-22, attenuate pathogen-induced tissue injury by enhancing antioxidant defenses, suppressing ROS accumulation, and inhibiting NLRP3 inflammasome activation, thereby reducing pro-inflammatory cytokine expression and apoptosis in blunt snout bream (*Megalobrama amblycephala*) [41]. Collectively, these findings establish the ROS–NLRP3 inflammasome axis as a central mechanism linking oxidative stress to inflammation and tissue damage in aquatic organisms.

A third mechanism through which oxidative stress exacerbates inflammation involves lipid peroxidation (LPO) [42]. ROS attack polyunsaturated fatty acids in cellular membranes, producing cytotoxic aldehyde byproducts such as malondialdehyde (MDA) and 4-hydroxynonenal (4-HNE). These reactive aldehydes disrupt membrane integrity, trigger cell lysis, and release intracellular contents, including proteases, nucleic acids, and oxidized proteins, that act as secondary DAMPs. These molecules further recruit macrophages and neutrophils to the injury site, which in turn release additional inflammatory cytokines and ROS, forming a vicious cascade of oxidative damage, immune cell recruitment, and tissue injury [43]. Collectively, available evidence suggests that excessive ROS production exacerbates oxidative stress and inflammation in aquatic animals by inducing lipid peroxidation and compromising antioxidant defenses; however, the detailed molecular mechanisms and species-specific regulatory pathways underlying this process remain to be further elucidated.

### 3.2. Inflammation as a Downstream Amplifier of Oxidative Stress

Prolonged exposure to inflammatory stimuli can induce metabolic reprogramming and oxidative imbalance, thereby forming a feedback loop that amplifies oxidative stress. Once initiated, inflammation can further intensify oxidative stress, creating a downward spiral that reinforces tissue damage. Activated immune cells, particularly macrophages and neutrophils, generate large quantities of ROS through a process known as the respiratory burst. During this process, the membrane-bound NADPH oxidase (NOX) complex catalyzes the one-electron reduction of oxygen to superoxide anion (O_2_^−^). The resulting ROS serve as antimicrobial agents, contributing to pathogen killing through oxidative damage to bacterial membranes, proteins, and DNA. However, excessive or prolonged activation of this process leads to ROS spillover, causing collateral damage to host tissues and perpetuating oxidative stress [6,10,13]. This self-reinforcing mechanism has been well documented in aquatic models. In shrimp, intestinal macrophage activation triggers intense respiratory bursts, producing elevated ROS levels that compromise intestinal epithelial integrity. The expression of tight junction proteins such as occludin and claudin decreases markedly, increasing gut permeability and facilitating further pathogen invasion [44,45]. Similarly, sustained inflammatory signaling through TNF-α and IL-6 has been shown to suppress antioxidant defenses and promote oxidative imbalance in zebrafish (*Danio rerio*) [46].

Another important downstream effect of inflammation is the suppression of the antioxidant system. Proinflammatory cytokines, including IL-6 and TNF-α, can downregulate the transcription and enzymatic activity of antioxidant enzymes such as SOD, CAT, and GPx. They also deplete nonenzymatic antioxidants like glutathione (GSH), either by consumption in redox reactions or by inhibiting glutathione synthesis pathways [47]. In Nile tilapia, bacterial infection with *Clostridium perfringens* induced pronounced inflammatory responses characterized by elevated pro-inflammatory cytokines, which were accompanied by a marked reduction in antioxidant enzyme activities (SOD, CAT and GPx) and a persistent increase in lipid peroxidation, indicating inflammation-associated impairment of antioxidant defenses [6]. Similarly, low-temperature stress in common carp triggered systemic inflammation and oxidative damage along the intestine–hepatopancreas axis, leading to hepatopancreatic injury and metabolic dysregulation concomitant with disrupted redox homeostasis in common carp (*C. carpio*) [48]. Consistent with this notion, a high-fat diet induced hepatic inflammation and oxidative stress in common carp (*C. carpio*), significantly suppressing antioxidant capacity, whereas dietary resveratrol alleviated inflammation and restored antioxidant enzyme activities through activation of Nrf2 signaling and inhibition of NF-κB-mediated inflammatory pathways [37].

**Figure 1:**
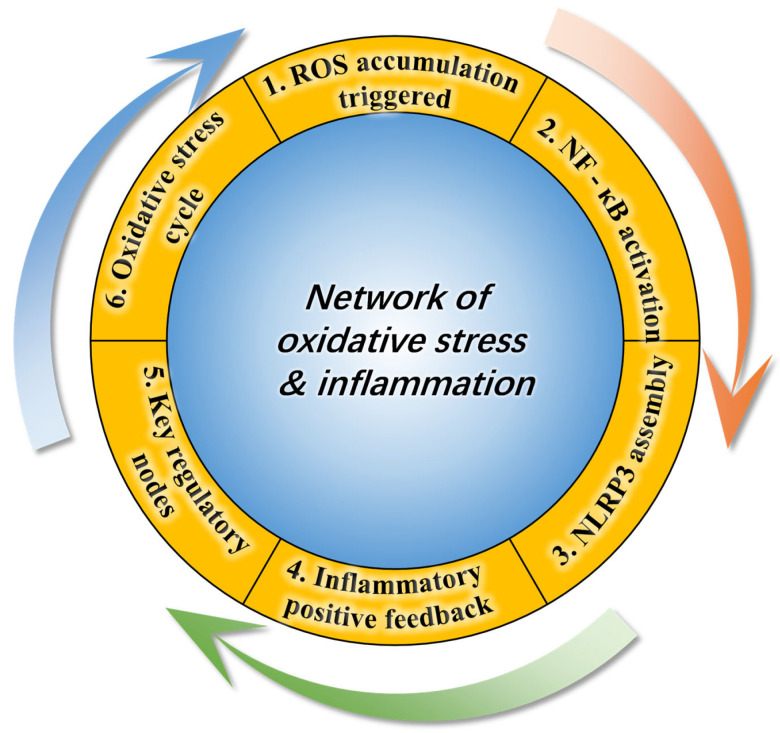
Bidirectional regulatory network between oxidative stress and inflammation via the NF-κB/NLRP3 pathway. The diagram depicts a vicious cycle in six steps: (1) ROS accumulation activates NF-κB and promotes NLRP3 inflammasome assembly, initiating inflammation; (2) IκB phosphorylation drives NF-κB nuclear translocation and inflammatory gene expression; (3) NLRP3 oligomerization activates caspase-1, leading to IL-1β and IL-18 maturation and release; (4) Inflammation induces mitochondrial damage, sustaining ROS production and amplifying oxidative stress; (5) Key regulatory nodes include IκB phosphorylation and NLRP3 assembly, controlling inflammatory intensity; (6) Mutual reinforcement between ROS and inflammation forms a malignant cycle underlying chronic inflammatory diseases.

Together, these findings establish a self-propagating loop in which oxidative stress activates inflammatory signaling, and inflammation, in turn, amplifies oxidative injury (Figure 1). This reciprocal relationship forms the molecular foundation for many stress-related pathologies observed in aquaculture species, including hepatic dysfunction, intestinal barrier disruption, and systemic immunosuppression. Breaking this cycle through dietary antioxidants, immunomodulatory agents, or environmental management strategies represents a promising direction for improving aquatic animal health and sustainability.

## 4. Biological Consequences: From Immune Imbalance to Increased Disease Susceptibility

The vicious cycle between oxidative stress and inflammation exerts profound biological effects that extend from cellular dysfunction to systemic immune imbalance, ultimately compromising the health and productivity of aquaculture species. This interconnected pathology manifests in several overlapping dimensions, including tissue injury, organ dysfunction, immune suppression, and a decline in stress resilience. Together, these effects directly determine disease susceptibility and influence the success of health management strategies in aquaculture systems. To illustrate these multifaceted consequences across diverse aquaculture species and stressors, Table 1 provides a comparative summary of representative inflammatory responses and corresponding alterations in antioxidant defenses, highlighting patterns observed in fish and crustaceans under environmental, nutritional, and pathogenic challenges. This table underscores how the vicious cycle manifests differently yet consistently across groups, reinforcing the need for targeted, mechanism-based interventions to mitigate tissue damage, organ dysfunction, and heightened disease risk in sustainable aquaculture practices.

### 4.1. Tissue Damage and Organ Dysfunction

The liver, being the primary metabolic and detoxification center, is highly vulnerable to lipid peroxidation triggered by ROS. The peroxidation of membrane lipids produces reactive aldehydes like MDA and 4-HNE, which form covalent adducts with proteins and nucleic acids, impairing hepatocellular integrity [49]. In Nile tilapia (*O. niloticus*), exposure to combined ammonia and salinity stress disrupted hepatic redox homeostasis, as evidenced by elevated lipid peroxidation (MDA) and altered antioxidant systems (reduced CAT activity and GSH/GSSG ratio), indicating oxidative stress-associated liver impairment [50]. Supporting evidence from other aquatic species further demonstrates that ammonia exposure induces coordinated alterations in hepatic morphology, antioxidant defenses, and immune function. For example, acute ammonia nitrogen stress in juvenile Chinese soft-shelled turtles (*Pelodiscus sinensis*) caused severe structural damage to the liver, characterized by disruption of hepatic cord architecture, vacuolation, and edema, accompanied by marked activation of antioxidant defenses (increased SOD and CAT activities and upregulated antioxidant gene expression) and dysregulation of ammonia metabolism. Prolonged exposure was associated with increased pathogen susceptibility, indicating compromised hepatic immune function [51]. Similarly, combined ammonia and nitrite exposure in largemouth bass (*Micropterus salmoides*) aggravated nitric oxide accumulation, suppressed antioxidant capacity, disturbed hepatic glucose and lipid metabolism, and triggered inflammatory responses, further confirming the close linkage between oxidative stress, antioxidant imbalance, liver dysfunction, and immune disturbance under ammonia stress [52]. In addition, *Aeromonas hydrophila* infection induced pronounced alterations in antioxidant enzyme activities (SOD, CAT and GPx) accompanied by severe histopathological damage, with the liver being the most affected organ in both Nile tilapia and common carp, highlighting infection-driven oxidative and inflammatory injury in key immune tissues [53]. Moreover, nutritional stress imposed by a high-fat diet led to hepatic lipid accumulation, inflammation, and structural damage in Nile tilapia, whereas dietary l-carnitine supplementation alleviated oxidative and inflammatory stress and partially restored liver histopathology and metabolic function [54].

The intestinal mucosa, serving as both a digestive and immune barrier, is another primary target of oxidative–inflammatory injury. Excessive ROS disrupt epithelial tight junction proteins such as occludin and claudin, weakening barrier integrity and allowing translocation of pathogenic bacteria. This not only results in local inflammation but also contributes to dysbiosis, characterized by an imbalance between beneficial and pathogenic microbial communities. In grass carp, a high-fat diet markedly impaired intestinal antioxidant capacity, down-regulated tight junction proteins, activated NF-κB-mediated inflammatory responses, and induced intestinal microbiota dysbiosis [55]. Consistently, nutritional imbalance caused by phenylalanine deficiency or excess disrupted the expression of tight junction proteins (ZO-1, occludin and claudins), suppressed antioxidant signaling, and activated pro-inflammatory cytokines through NF-κB and Nrf2 pathways, indicating a close linkage between oxidative stress, inflammation, and intestinal barrier dysfunction in grass carp (*Ctenopharyngodon idella*) [56]. Moreover, in Pacific white shrimp (*Litopenaeus vannamei*), ammonia and nitrite stress directly damaged intestinal mucosal structure, altered mucus immune gene expression, and induced pronounced dysbiosis characterized by increased opportunistic pathogens [57]. Similarly, chronic ammonia nitrogen exposure in Chinese soft-shelled turtles (*Pelodiscus sinensis*) compromised intestinal mechanical and chemical barriers by downregulating tight junction and mucus-related genes (Occludin, ZO-1, and MUC2), modulated NF-κB signaling, and induced dose-dependent alterations in gut microbial composition and interaction networks [58], further confirming that sustained ammonia stress disrupts intestinal barrier integrity and microbial homeostasis across aquatic species.

Gill tissues, essential for respiration and ion regulation, also experience structural degradation under oxidative–inflammatory stress. Elevated ROS damages epithelial cells, leading to lamellar fusion, epithelial lifting, and reduced gas exchange capacity. In *Cirrhinus mrigala*, exposure to the herbicide formulation Bromoxynil and MCPA (2-Methyl-4-chlorophenoxyacetic acid) induced pronounced oxidative stress characterized by elevated ROS and lipid peroxidation alongside suppressed antioxidant enzyme activities, which were accompanied by severe gill histopathological lesions, including epithelial uplifting and lamellar fusion [59]. Similarly, nutritional stress caused by pyridoxine deficiency in grass carp (*C. idella*) resulted in excessive ROS accumulation, reduced antioxidant capacity, activation of NF-κB-mediated inflammation, and downregulation of tight junction proteins, thereby compromising gill barrier integrity and increasing susceptibility to epithelial damage [60]. Conversely, mitigation of oxidative stress effectively preserved gill structure, as evidenced in razor clams, where exogenous proline supplementation enhanced antioxidant defenses, reduced ROS and MDA levels, and prevented gill filament damage under hypersalinity stress [61].

**Figure 2:**
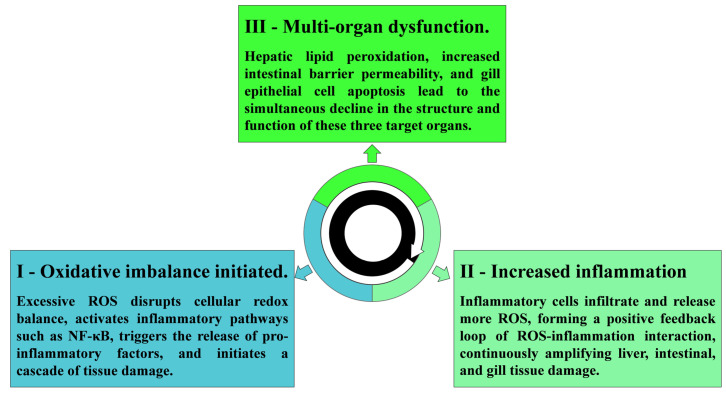
Multi-organ functional impairments induced by the oxidative stress-inflammation bidirectional network. The figure outlines three major pathological outcomes: (1) Oxidative stress initiation: Excess ROS disrupts redox balance, activates NF-κB, triggers cytokine release, and initiates inflammatory cascades; (2) Inflammation positive feedback: Infiltrating immune cells release additional ROS, forming a self-amplifying loop that exacerbates tissue damage; (3) Multi-organ damage: Impaired lipid metabolism causes hepatic steatosis, disrupted intestinal barrier increases permeability, and apoptosis affects hepatocytes and intestinal epithelium, leading to synchronous dysfunction of liver, intestine, and gill.

Oxidative stress and chronic inflammation lead to progressive structural damage and loss of function in key immune and metabolic organs such as the liver, intestine, and gills (Figure 2). Collectively, damage to critical organs such as the liver, intestine, and gills in aquatic animals disrupts metabolic regulation, epithelial barrier integrity, and respiratory and osmoregulatory functions. These organ-specific impairments weaken immune defenses and facilitate pathogen invasion under environmental, nutritional, or pathogenic stress conditions commonly encountered in aquaculture systems. Ultimately, multi-organ dysfunction compromises physiological stability, reduces disease resistance, and undermines the capacity of aquatic animals to maintain homeostasis.

### 4.2. Immune Suppression and Increased Pathogen Susceptibility

Chronic oxidative stress and sustained inflammatory activation profoundly impair immune function in aquatic animals by directly damaging immune cells. In Nile tilapia (*O. niloticus*), exposure to a wide range of immuno-toxic chemicals consistently suppressed cytotoxic T lymphocyte activity, indicating a marked reduction in immune cell–mediated cytolytic capacity and highlighting the vulnerability of cellular immunity to chronic chemical stress [62]. Similarly, long-term cadmium exposure in common carp induced excessive ROS accumulation and mitochondrial dysfunction in gill tissues, triggering apoptosis through mitochondria- and JNK–FoxO3a–PUMA-dependent pathways, while simultaneously disrupting Th17/Treg immune balance, ultimately resulting in pronounced immunosuppression [63]. Conversely, attenuation of oxidative and inflammatory stress effectively preserved immune function, as dietary α-lipoic acid supplementation mitigated cold stress-induced oxidative damage, suppressed pro-inflammatory and pro-apoptotic signaling, and restored antioxidant enzyme expression and immune competence in tilapia [64]. These studies demonstrate that ROS-driven oxidative–inflammatory stress compromises immune cell survival and function, thereby potentially compromising defense capacity of aquatic animals.

A particularly concerning consequence of oxidative–inflammatory imbalance is the increased susceptibility of aquatic animals to viral and bacterial infections, as the feedback loop between oxidative stress and inflammation can intensify disease severity and lethality. Multiple studies demonstrate that restoration of antioxidant and immune homeostasis markedly enhances disease resistance, indirectly highlighting the detrimental role of oxidative–inflammatory dysregulation. In abalone (*Haliotis discus hannai*), excessive oxidative stress and inflammatory activation were associated with reduced antioxidant enzyme activity, suppressed immune gene expression, and increased mortality following *Vibrio parahaemolyticus* challenge [65]. Similarly, in common carp (*C. carpio*), dietary cornelian cherry extract enhanced antioxidant capacity and immune responses, resulting in markedly improved survival following *Aeromonas hydrophila* infection [66]. In Nile tilapia (*O. niloticus*), licorice root powder supplementation strengthened antioxidant defenses and innate immune parameters and dramatically increased post-challenge survival, while control fish with weaker redox and immune status exhibited the highest susceptibility to bacterial infection [67]. Consistently, probiotic-fortified brown seaweed diets enhanced mucosal barrier integrity, antioxidant capacity, and anti-inflammatory signaling in barramundi, leading to substantially reduced mortality after *V. harveyi* challenge [68]. Collectively, these findings indicate that oxidative–inflammatory imbalance predisposes fish and shellfish to heightened pathogen susceptibility, whereas disruption of this feedback loop effectively attenuates disease virulence and mortality.

At the molecular level, ROS-mediated oxidation of immune receptors and cytokine regulators can alter signal transduction, further aggravating immune dysfunction. Persistent NF-κB and NLRP3 activation not only perpetuate inflammation but also exhaust immune resources and induce tolerance-like states, thereby diminishing responsiveness to subsequent infections. This dysregulated immune landscape mirrors immunosenescence, a state of functional decline in immune capacity, observed in aged or chronically stressed aquatic animals [31,35,39,40].

### 4.3. Reduced Stress Adaptation and Increased Metabolic Costs

#### 4.3.1. Environmental Stress as a Primary Driver of ROS Overproduction

**Figure 3:**
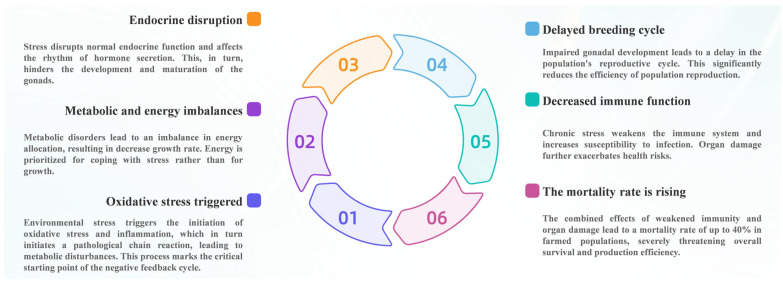
Vicious cycle of chronic stress-induced negative impacts in farmed animals.

Environmental stressors commonly encountered in aquaculture systems, including hypoxia [69], temperature fluctuation [70,71], salinity variation [72], high ammonia [73] or nitrite [74] levels, and crowding [75], are well-recognized triggers of cellular oxidative stress. At the cellular level, generalized stress responses are accompanied by increased energy demand and elevated metabolic activity, particularly within mitochondria [76]. Enhanced electron flux through the respiratory chain under stress conditions increases the probability of electron leakage and results in excessive mitochondrial ROS production [77]. When ROS generation exceeds the buffering capacity of antioxidant systems, oxidative stress ensues, initiating redox imbalance and activating downstream inflammatory signaling pathways. Thus, environmental stressors should be regarded as upstream initiating factors in the oxidative stress–inflammation feedback cycle (Figure 3), acting at the earliest stage by promoting mitochondrial ROS overproduction and redox disturbance.

The maintenance of redox homeostasis, repair of damaged biomolecules, and persistent immune activation require high ATP expenditure, diverting energy from essential physiological processes such as growth, reproduction, and environmental adaptation. In rainbow trout (*Oncorhynchus mykiss*), environmental hypoxia significantly reduced oxygen consumption, feed intake, nutrient digestibility, and energy retention, while increasing maintenance energy requirements and heat production, indicating that sustained stress diverts energy away from growth and body energy storage toward basic survival and physiological maintenance [78]. Similarly, in Pacific white shrimp (*L. vannamei*), combined high salinity and ammonia-nitrogen stress disrupted glucose metabolism, enhanced anaerobic metabolism, suppressed nonspecific immune responses, and altered antioxidant defense systems, reflecting a reallocation of metabolic energy toward stress resistance, detoxification, and redox regulation at the expense of immune competence and overall physiological performance [79].

#### 4.3.2. Oxidative–Inflammatory Stress Drives Energy Reallocation and Endocrine Disruption

Once oxidative stress and inflammation are established, they impose substantial energetic and metabolic burdens on aquatic animals. The maintenance of redox homeostasis, repair of oxidatively damaged biomolecules, and sustained activation of immune responses require continuous ATP investment. As a consequence, metabolic energy is preferentially redirected away from growth, reproduction, and long-term adaptation toward basic survival processes, detoxification, and immune defense. Chronic oxidative–inflammatory stress also disrupts endocrine and neuroendocrine regulation of stress adaptation. Persistent activation of the hypothalamic–pituitary–interrenal (HPI) axis elevates circulating cortisol and catecholamines, which further exacerbate oxidative pressure by stimulating mitochondrial ROS generation while simultaneously suppressing antioxidant enzyme activity. This hormonal imbalance weakens physiological resilience to additional environmental challenges and increases the metabolic cost of maintaining homeostasis. Ultimately, the combined effects of energy depletion, endocrine dysregulation, and immune suppression reduce stress tolerance and disease resistance at the organismal level. Breaking the oxidative–inflammatory loop through antioxidant nutrition (e.g., selenium, astaxanthin, vitamin E), immunomodulatory probiotics, and optimized rearing environments has therefore become a key focus in contemporary aquaculture health management [16,21,22,23].

**Figure 4:**
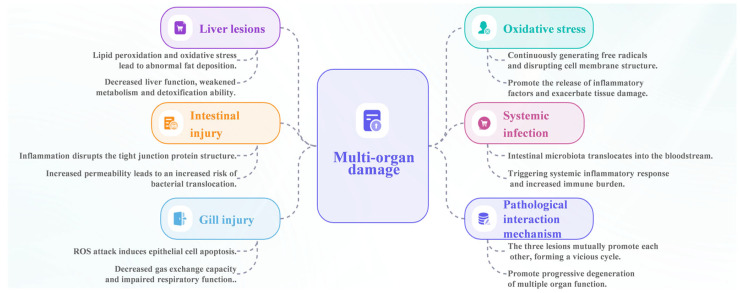
Pathological interaction mechanism leading to multi-organ damage. The figure displays interconnected organ-specific injuries resulting in systemic multi-organ damage: liver lesions (lipid peroxidation and fat deposition), intestinal injury (disrupted tight junctions and bacterial translocation), gill injury (epithelial apoptosis and impaired respiration), oxidative stress (free radical generation and inflammation), systemic infection (microbiota translocation), and pathological interactions that mutually reinforce each other, forming a vicious cycle of progressive organ dysfunction.

In summary, environmental stressors act as primary initiators of oxidative stress, triggering mitochondrial ROS overproduction at the cellular level (Figure 3). The resulting oxidative stress activates inflammatory responses, which in turn exacerbate redox imbalance, energy depletion, endocrine disruption, and immune incompetence. This self-reinforcing cycle progressively erodes stress adaptability and physiological performance, thereby increasing disease susceptibility and undermining aquaculture sustainability (Figure 4). Breaking this cycle through optimized environmental management, antioxidant nutrition, and immune-supportive strategies is therefore essential for maintaining metabolic efficiency, stress resilience, and animal welfare in intensive aquaculture systems.

## 5. Applied Insights for Aquaculture: Mechanism-Based Strategies for Disease Prevention and Health Management

**Figure 5:**
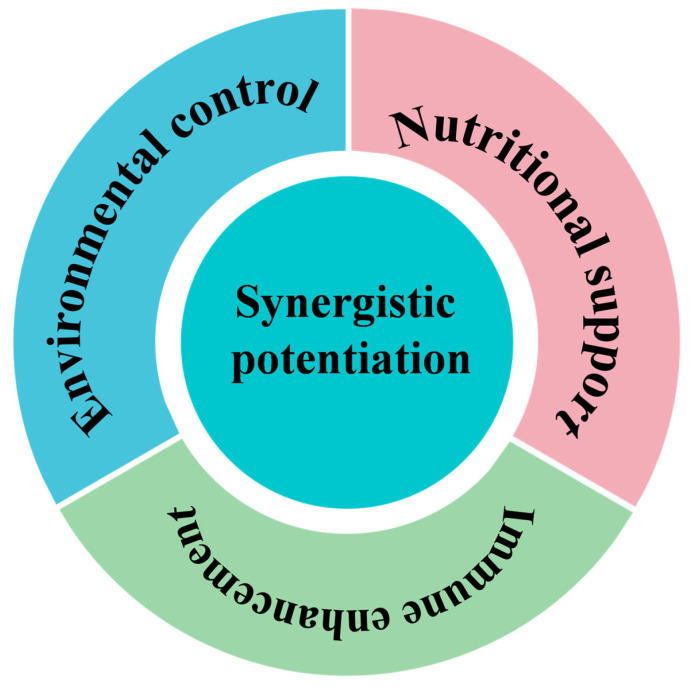
Environment–nutrition–immunity integrated three-in-one defense system. The schematic outlines a comprehensive preventive strategy comprising four key components: environmental regulation (optimizing water parameters to reduce external stressors), nutritional support (providing precise antioxidative nutrition to enhance defense), immune enhancement (stimulating innate immunity to improve disease resistance), and synergistic efficacy (integrating the three elements into a proactive, dynamic health management approach).

The bidirectional regulation between oxidative stress and inflammation highlights the need for integrated, mechanism-based disease prevention strategies in aquaculture. Traditional approaches have often emphasized either antioxidant supplementation or anti-inflammatory modulation alone; however, such single-target interventions overlook the dynamic feedback between redox imbalance and immune activation. To effectively mitigate stress-induced pathologies and enhance resilience in cultured species, a multi-layered strategy is required, one that combines source mitigation, loop interruption, and immune reinforcement (Figure 5). This integrative framework aims to suppress the initiation of oxidative stress, block the propagation of inflammatory amplification, and strengthen host defense capacity, ultimately achieving sustainable health management in aquaculture systems.

### 5.1. Reducing Oxidative Stress at the Source

The first step in controlling oxidative and inflammatory cascades is to minimize exposure to environmental and metabolic stressors that trigger ROS overproduction. Environmental stress refers to external factors, such as changes in water temperature, oxygen levels, salinity, and pollutant concentrations, which disrupt an organism’s homeostasis. These stressors can have wide-ranging effects on physiological systems, including metabolism. Metabolic stress, on the other hand, refers to internal physiological disruptions that occur when the organism’s metabolic processes are disturbed. This type of stress arises from factors like nutrient imbalances, hormonal disruptions, or extreme temperature conditions that compromise cellular function. In essence, environmental stress is a major contributor to metabolic stress, as it often leads to changes in metabolism, triggering oxidative stress, and disrupting redox balance. To prevent oxidative stress, environmental optimization is essential. Proper regulation of stocking density, maintenance of adequate dissolved oxygen levels, and control of ammonia and nitrite concentrations are critical for reducing mitochondrial electron leakage and respiratory chain dysfunction, both of which are major sources of ROS. In Pacific white shrimp (*L. vannamei*), alleviation of ammonia and nitrite stress was associated with attenuation of oxidative damage and recovery of antioxidant defenses, including increased activities of SOD and CAT, indicating improved redox homeostasis following stress relief [57]. Similarly, studies involving ammonia-related environmental stress demonstrated that lowering ammonia burden mitigated oxidative pressure and restored antioxidant capacity, as evidenced by reduced lipid peroxidation and normalization of antioxidant enzyme systems in aquatic species [79].

Nutritional optimization plays another crucial role in mitigating oxidative stress. Adequate intake of antioxidants and redox-regulating compounds provides biochemical support to neutralize ROS and stabilize cellular redox homeostasis. In Nile tilapia (*Oreochromis niloticus*), dietary supplementation with α-lipoic acid, a potent antioxidant and glutathione-regenerating molecule, significantly enhanced antioxidant capacity, restored the expression of key antioxidant enzymes (SOD and CAT), and attenuated oxidative damage, inflammation, and apoptosis under cold stress conditions [64]. Moreover, ALA supplementation alleviated stress-induced histopathological damage in the liver, spleen, and intestine while improving immune and metabolic performance, underscoring the importance of antioxidant nutritional support in maintaining redox balance and physiological integrity in aquatic animals. Natural plant-derived antioxidants, such as artichoke leaf extract, have also gained attention for their dual role in scavenging ROS and modulating immune signaling. In Nile tilapia, for instance, dietary artichoke leaf extract alleviated fluoride-induced oxidative damage by restoring antioxidant defenses and suppressing pro-inflammatory cytokine expression, supporting the immunomodulatory and antioxidative potential of plant-derived compounds [5]. By combining environmental regulation with nutritional reinforcement, aquaculture practitioners can effectively suppress the upstream triggers of oxidative stress and prevent its initiation.

### 5.2. Interrupting the Vicious Cycle: Coordinated Antioxidant and Anti-Inflammatory Regulation

The second layer of intervention focuses on disrupting the self-reinforcing oxidative–inflammatory loop through targeted regulation of molecular pathways. Instead of relying on single-function agents, recent research emphasizes the synergistic use of compounds possessing both antioxidant and anti-inflammatory activities. Such dual-function substances act at the interface of redox and immune signaling, simultaneously scavenging ROS and attenuating proinflammatory gene expression.

Curcumin is a well-documented example, capable of directly scavenging hydroxyl radicals (•OH) while inhibiting NF-κB activation and COX-2 expression. For example, curcumin treatment markedly enhanced antioxidant enzyme activities, reduced oxidative damage, and suppressed the expression of NF-κB-dependent pro-inflammatory cytokines, indicating that curcumin alleviates oxidative and inflammatory stress through activation of Nrf2 signaling and inhibition of the NF-κB pathway in grass carp (*C. idella*) after injection with *A. hydrophila* [80] and koi carp (*Cyprinus carpio haematopterus*) following koi herpesvirus infection [81]. Similarly, astaxanthin provides another representative example of a natural antioxidant with dual antioxidant and immunomodulatory functions. In largemouth bass (*M. salmoides*) fed a high-carbohydrate diet, astaxanthin supplementation effectively attenuated oxidative stress, inflammation, and apoptosis by activating the Keap1–Nrf2–ARE pathway while suppressing NF-κB-mediated inflammatory signaling, thereby alleviating immunological disorders and tissue damage in an organ-specific manner [82].

A critical consideration in this stage is avoiding excessive immune stimulation. While immunostimulants and vaccines are indispensable for disease prevention, their inappropriate use can paradoxically trigger overactive inflammatory responses. Overdosage of LPS-like adjuvants, for example, induces uncontrolled macrophage activation and respiratory bursts, leading to elevated ROS generation and collateral tissue injury of zebrafish [31]. Controlled immunization protocols with precise dosing and timing are therefore essential. In rainbow trout (*Oncorhynchus mykiss*), injection with inactivated *V. anguillarum* bacteria induced early cortisol and metabolic stress responses followed by delayed upregulation of pro-inflammatory cytokines, highlighting the need for precise immunization strategies to avoid excessive stress and inflammation [83]. The balance between immune activation and redox stability must thus be carefully maintained to achieve effective yet safe immune protection.

### 5.3. Targeting Viral Diseases: Breaking the ROS–Inflammation–Virus Replication Loop

Viral infections in aquaculture species present a unique challenge, as they are tightly linked to oxidative and inflammatory pathways. ROS not only mediate tissue injury during viral infection but also actively facilitate viral replication by modulating host signaling cascades. In grass carp, Grass Carp Reovirus (GCRV) infection induces endoplasmic reticulum stress and activates the PERK–eIF2α pathway, leading to enhanced ROS production via the PERK–ERO1α axis, which in turn promotes viral replication [84]. Consistently, modulation of host redox status also affects viral propagation, as SOD reduce intracellular ROS levels, suppress autophagy, and thereby facilitate GCRV replication, whereas ROS elevation restrains viral replication through autophagy activation [85].

Breaking the ROS–inflammation–virus replication feedback loop requires a combination of antioxidant and immunomodulatory strategies. Early-phase intervention using potent antioxidants such as GSH, vitamin E, or NAC can suppress ROS accumulation and hinder viral replication by disrupting redox-dependent viral transcriptional control. Concurrent administration of anti-inflammatory cytokines, such as recombinant IL-10 or IL-4, can attenuate tissue-damaging inflammation without impairing antiviral defense. Oral immunization of grass carp with recombinant *Lactobacillus casei* expressing the GCRV VP5 antigen significantly upregulated antiviral immune responses, reduced viral loads, and markedly improved survival following GCRV challenge, demonstrating that appropriately designed immunomodulatory interventions can effectively suppress viral replication while maintaining immune balance [86]. This dual-protection framework aligns with the emerging concept of redox-immunological balance in aquaculture, which recognizes oxidative–inflammatory equilibrium as a key determinant of viral pathogenesis and host tolerance. The strategic combination of antioxidants, anti-inflammatory modulators, and vaccines therefore represents a forward-looking approach for controlling viral diseases and safeguarding aquaculture sustainability.

Collectively, the evidence reviewed in this article demonstrates that oxidative stress and inflammation are not independent pathological events in aquatic animals, but rather form a tightly coupled, self-amplifying network driven by reciprocal regulation of redox imbalance and immune activation. Key signaling pathways, including PERK–eIF2α–ERO1α–ROS, JNK–FoxO3a–PUMA, and the Nrf2/NF-κB axis, function as molecular nodes that translate environmental and nutritional stressors into oxidative damage, inflammatory escalation, and tissue dysfunction. From a practical aquaculture perspective, these insights underscore that effective disease prevention cannot rely on single-target antioxidant or anti-inflammatory measures alone.

Instead, a mechanism-guided, multi-level health management strategy is required. At the production level, priority should be given to reducing stress at the source, through optimization of water quality parameters (e.g., ammonia, nitrite, dissolved oxygen) and rational stocking density, thereby limiting excessive ROS generation upstream. At the nutritional level, feeds should be designed to include dual-function additives that simultaneously reinforce antioxidant defenses and restrain inflammatory signaling. Natural compounds such as curcumin, astaxanthin, α-lipoic acid, and selected plant-derived extracts represent practical examples, as they modulate Nrf2-dependent antioxidant pathways while suppressing NF-κB-mediated inflammation, effectively interrupting the oxidative–inflammatory vicious cycle.

Importantly, immune-based interventions must be applied with precision. While vaccines, immunostimulants, and probiotics are indispensable tools for disease control, inappropriate dosage or timing can provoke excessive inflammatory responses and secondary oxidative injury. Mechanistic understanding of stress-activated pathways (e.g., PERK–eIF2α–ROS during viral infection or JNK-driven apoptosis under chronic inflammation) provides a rational framework for optimizing immunization strategies that maximize protection while minimizing collateral damage.

Taken together, these findings offer aquaculture producers a clear conceptual guideline: health management should integrate environmental regulation, functional nutrition, and controlled immune modulation, rather than treating oxidative stress or inflammation as isolated targets. Such an integrated strategy shifts disease control from a reactive approach toward proactive resilience enhancement. Future advances in precision nutrition, biomarker-guided management, and selective breeding targeting redox–immune regulators (e.g., Nrf2, Keap1, NF-κB) will further enable the translation of molecular mechanisms into sustainable aquaculture practice.

## 6. Conclusions

Oxidative stress and inflammation are tightly interconnected processes that together define the core pathophysiological framework of aquatic animal health. At the mechanistic level, ROS act as pivotal signaling molecules linking oxidative damage to immune activation, forming a self-perpetuating feedback loop in which oxidative stress triggers inflammation, and inflammation in turn amplifies ROS generation. This bidirectional amplification disrupts immune homeostasis, impairs tissue integrity, and heightens disease susceptibility in cultured species. Importantly, overall stress, including environmental, nutritional, and pathological stressors, initiates this oxidative stress–inflammation loop, exacerbating the detrimental effects on aquatic health. Understanding this underlying logic provides a unifying perspective for interpreting stress adaptation, immune imbalance, and disease progression in aquaculture. Effective control, therefore, must move beyond isolated antioxidant or anti-inflammatory strategies toward integrated approaches that concurrently reduce ROS production, reinforce antioxidant capacity, and prevent excessive inflammatory activation.

Future research and disease management in aquaculture should focus on breaking the ROS–inflammation–virus replication loop that drives many viral and stress-related disorders. Integrating antioxidants, anti-inflammatory modulators, and immune enhancers offers a promising pathway for maintaining redox–immune balance. The development of multifunctional compounds, capable of both scavenging ROS and modulating immune signaling, will be key to improving resilience and reducing mortality. Moreover, emerging technologies such as precision nutrition, omics-guided breeding, and the design of synthetic or recombinant antioxidant–anti-inflammatory–immune-enhancing molecules hold great potential to transform disease prevention. By targeting core regulatory nodes such as Nrf2, NF-κB, and MAPK, future strategies can shift aquaculture health management from reactive treatment to proactive physiological regulation, achieving sustainable productivity and enhanced animal welfare.

## Figures and Tables

**Table 1:** Summary of key inflammatory responses and affected antioxidant defenses in aquatic animals under various environmental, nutritional, and pathogenic stress factors.

Species	Stress Factor	Inflammatory Responses	Affected Antioxidant Defenses	Reference
*Ctenopharyngodon idella*	Nutritional deficiency (e.g., phenylalanine)	Activation of NF-κB; elevated pro-inflammatory cytokines (IL-1β, IL-8, TNF-α); disruption of epithelial barrier integrity	Elevated ROS; impaired antioxidant capacity; downregulation of Nrf2 signaling; reduced SOD, CAT, GPx activities	[34]
*Carassius gibelio*	Pathogenic challenge (LPS-induced)	Hepatic inflammation; NF-κB activation; increased pro-inflammatory mediators	ROS accumulation; oxidative damage; suppressed antioxidant enzymes (alleviated by resveratrol via Nrf2)	[36]
*Cyprinus carpio*	Environmental pollutant (difenoconazole exposure)	Gill inflammation; NF-κB–NLRP3 inflammasome activation; pyroptosis; elevated IL-1β	ROS accumulation; lipid peroxidation (MDA increase); reduced SOD, CAT, GPx; restored by antioxidants like ferulic acid	[37]
*Larimichthys crocea*	Nutritional stress (excessive palmitic acid)	NLRP3 inflammasome activation via NF-κB; impaired AMPK-mediated mitophagy; IL-1β–mediated inflammation	Mitochondrial dysfunction; ROS overproduction; oxidative imbalance	[40]
*Megalobrama amblycephala*	Pathogenic infection (e.g., *Aeromonas hydrophila*)	Tissue injury; NLRP3 inflammasome activation; pro-inflammatory cytokine expression (e.g., IL-1β); apoptosis	ROS accumulation; suppressed antioxidant defenses; alleviated by IL-22 enhancing redox homeostasis	[41]
*Oreochromis niloticus*	Bacterial infection (*Clostridium perfringens*)	Elevated pro-inflammatory cytokines; systemic immune suppression	Reduced SOD, CAT, GPx activities; persistent lipid peroxidation (MDA increase)	[6]
*Cyprinus carpio*	Temperature stress (low-temperature)	Systemic inflammation along intestine–hepatopancreas axis; hepatopancreatic injury	Disrupted redox homeostasis; oxidative damage; metabolic dysregulation	[48]
*Cyprinus carpio*	Nutritional stress (high-fat diet)	Hepatic inflammation; NF-κB activation	Suppressed antioxidant capacity; alleviated by resveratrol activating Nrf2	[37]
*Danio rerio*	Environmental toxin (cyano-bacterial aphantoxins)	Brain inflammation; compensatory immune activation	Elevated ROS and MDA; depleted GSH; activated SOD, CAT, GPx	[18]
*Litopenaeus vannamei*	Environmental stress (cold shock and air exposure)	Intestinal macrophage activation; respiratory burst; decreased tight junction proteins (occludin, claudin); increased gut permeability	Transient ROS accumulation; elevated MDA; tissue-specific antioxidant responses in hepatopancreas	[19]
*Litopenaeus vannamei*	Pathogen invasion (e.g., *Vibrio parahaemolyticus*)	Recruitment of macrophages/neutrophils; cytokine release (e.g., TNF-α, IL-6); tissue necrosis	ROS spillover from respiratory burst; suppressed antioxidant enzymes (SOD, CAT, GPx); GSH depletion	[44]

## Data Availability

No new data were created or analyzed in this study. Data sharing is not applicable to this article.
